# Water as a Source of Indoor Air Contamination with Potentially Pathogenic *Aeromonas hydrophila* in Aquaculture

**DOI:** 10.3390/ijerph19042379

**Published:** 2022-02-18

**Authors:** Iwona Gołaś, Mariusz Szmyt, Katarzyna Glińska-Lewczuk

**Affiliations:** 1 Department of Water Protection Engineering and Environmental Microbiology, University of Warmia and Mazury in Olsztyn, Prawocheńskiego 1, 10-720 Olsztyn, Poland; 2 Department of Ichthyology and Aquaculture, University of Warmia and Mazury in Olsztyn, Oczapowskiego 5, 10-719 Olsztyn, Poland; mariusz.szmyt@uwm.edu.pl; 3 Department of Water Management and Climatology, University of Warmia and Mazury in Olsztyn, Plac Łodzki 2, 10-719 Olsztyn, Poland; kaga@uwm.edu.pl

**Keywords:** indoor air, aquaculture laboratory, potentially pathogenic *Aeromonas hydrophila*, MAR index

## Abstract

Human activities influence the presence of potentially pathogenic bacteria in indoor air. The aim of this study was to determine the effect of the experimental rearing of European grayling and European perch in a recirculating aquaculture system on the contamination of indoor air with potentially pathogenic *Aeromonas hydrophila* (PPAH) and the resulting health risks to humans. The PPAH counts, their resistance to seven antibiotics, and the multiple antibiotic resistance (MAR) index were determined in samples of indoor air and water from rearing tanks. The PPAH counts were highest in the laboratory bioaerosol where two fish species were reared. The calculated indoor/outdoor ratio (I/O > 1) demonstrated that tank water was the internal source of PPAH emissions. The unconstrained PCA revealed strong positive relationships (*p* ≤ 0.05) between the PPAH counts in the indoor air and water samples. Most of the PPAH strains isolated from laboratory air were resistant to tetracycline, cefotaxime, and erythromycin, and 26–82% of the isolates exhibited multiple drug resistance. The values of the MAR index were similar in samples of laboratory air and water (0.23–0.34 and 0.24–0.36, respectively). Agglomerative clustering revealed two clusters of strains isolated from laboratory air and tank water. The results of this study indicate that aquaculture can be a source of indoor air contamination with PPAH.

## 1. Introduction

Indoor air is a highly dynamic system where particles of biological and non-biological origin are distributed and displaced. The microbiological quality and safety of indoor air (IAQ) affect human health. The above also applies to research laboratories, where scientists and technical personnel work for several to more than ten hours per day. During a single working day, employees breathe in around 10 m^3^ of air, and 10^6^ microbial cells reach their lungs [1,2]. Indoor air pollution has been ranked as one of the top five risks to public health by the United States Environmental Protection Agency (US EPA) [3]. Bioaerosol particles are one of the main causes of poor air quality, and their content in indoor air can be as high as 34% [4]. Bioaerosol particles are formed by liquid droplets and solid particles suspended in air, and they can contain bacteria, fungi, viruses, and their metabolites. Bioaerosols pose a serious health threat for humans because airborne bacteria and fungi can cause respiratory and digestive tract infections, as well as infections of the skin, eyes, and ears. The indoor bioaerosol levels are largely determined by the relative humidity, temperature, the outdoor concentrations of bioaerosols, and air exchange rates. Human activities and animal rearing can be a source of indoor contamination with microbial species that are predominant in bioaerosols [2,5,6]. 

According to the literature, potentially pathogenic microbiota in offices [7,8], educational facilities [9,10], hospitals [11,12], museums [13], and recreational facilities [14] pose health risks to the occupants. However, the microbiological quality and epidemiological safety of indoor air in research laboratories have not been assessed to date. To fill in this knowledge gap, indoor air was analyzed in a laboratory inside an experimental aquaculture facility. An attempt was made to evaluate the health risks associated with the presence of potentially pathogenic *Aeromonas hydrophila* (PPAH). These autochthonous bacteria constitute natural fish microbiota; they are ubiquitous in the aquatic environment and in fish rearing tanks, and they are one of the most commonly diagnosed epizootic risk factors in fish populations around the world [15,16,17]. Some *A. hydrophila* strains are also pathogenic for humans, and contaminated water, soil, and air are potential sources of infection [18,19]. In a study of zoonotic diseases associated with fish, Lehane and Rawlin [20] observed that fish pathogens caused cellulitis, myositis, and septicemia in aquaculture employees and home aquarium owners who suffered from injuries. In humans, clinical symptoms of *A. hydrophila* infections include skin and soft tissue infections, gastroenteritis, meningitis, and septicemia [21,22]. 

In view of the cyclic and specific nature of the research conducted by the employees and students of the University of Warmia and Mazury in Olsztyn (Poland), in the laboratory of the Aquaculture and Environmental Engineering Center, the aim of this study was to determine the effect of the experimental rearing of two fish species in a recirculating aquaculture system (RAS) on the contamination of indoor air with potentially pathogenic *Aeromonas hydrophila* (PPAH) present in water tanks and the resulting health risks to humans. The research hypothesis was validated by: (i) determining the counts of PPAH and their proportion in the total counts of mesophilic *A. hydrophila* (TCMAH); (ii) identifying the sources of PPAH and TCMAH emissions in indoor air by calculating the indoor/outdoor (I/O) ratios of pathogens; and (iii) calculating the multiple antibiotic resistance (MAR) index of the analyzed PPAH isolates to determine potential health risks for humans. 

## 2. Materials and Methods

### 2.1. Study Site

Indoor air was analyzed in the experimental facilities of the Aquaculture and Environmental Engineering Center of the University of Warmia and Mazury in Olsztyn, Poland. The building has a floor area of 2500 sq. m., and it was commissioned for use in 2011. The walls are made of aerated concrete blocks and glass. The building is mechanically ventilated, and it does not have a natural ventilation system enabling the flow of air between the outside and the inside. Research laboratories occupy 70% of the building’s area. In the laboratories, various fish species are reared and farmed, and experiments are conducted to optimize their reproductive performance and analyze the nutritional value of fish as a source of healthy food.

### 2.2. Research Premises and Sample Collection

The indoor air was sampled in two rooms located on the ground floor of the building. The first room was the laboratory (L), where European grayling (*Thymallus thymallus* L.) and European perch (*Perca fluviatilis*) were experimentally reared in RAS. The laboratory has a floor area of 41 m^2^ and a cubic capacity of 163 m^3^. The laboratory is equipped with a mechanical ventilation system with 8–10 air changes per hour according to ASHRAE standards [23]. The RAS in the laboratory consisted of 9 fish rearing tanks, a microstrainer, a trickling filter, and a UV lamp [24]. European grayling were reared between 15 April 2016 and 15 June 2016, and European perch—between 19 April 2019 and 19 June 2019. During the experiments, the water temperatures in the rearing tanks were determined to be 11.0 ± 0.2 °C (European grayling) and 18.0 ± 0.5 °C (European perch). In each experiment, 9 rearing tanks were stocked with 105 European grayling (total biomass: 52.3 ± 1.8 kg) and 120 European perch (total biomass: 38.6 ± 2.3 kg). Both fish species were administered commercial feed ad libitum twice a day. The fish were fed and handled by two employees who spent 2 to 4 h per day in the laboratory. Antibiotics were not administered to the fish during the experiments. After the experiment, the water was removed from the RAS, and all system components were disinfected with 0.5% Steridial solution according to the procedure described by Terech-Majewska [25]. The air inside the laboratory was sterilized with a UV lamp emitting light with a wavelength of 230–280 nm. 

The second room was the hallway (H) connecting the laboratory to the main building hall (MH), and the air was sampled around 10 m from the laboratory entrance. The hallway has a floor area of 50 m^2^ and a cubic capacity of 125 m^3^. The hallway is not ventilated, and it leads to offices on the first floor. Each day, the hallway was used by 10–15 persons on average. 

Control samples of outdoor air (C) were collected at a distance of 5 m from the building outside the zone of exposure to bioaerosols present in the laboratory (L) and the hallway (H). The outdoor air was sampled to determine the background levels of microbial contamination and potential migration of the analyzed microorganisms to indoor facilities. 

To verify the research hypothesis, the counts of potentially pathogenic *A. hydrophila* (PPAH) and their proportion in the total counts of mesophilic *A. hydrophila* (TCMAH) were also monitored in the samples of water collected from the experimental rearing tanks stocked with two fish species with different temperature requirements: European grayling and European perch. 

**Figure 1:**
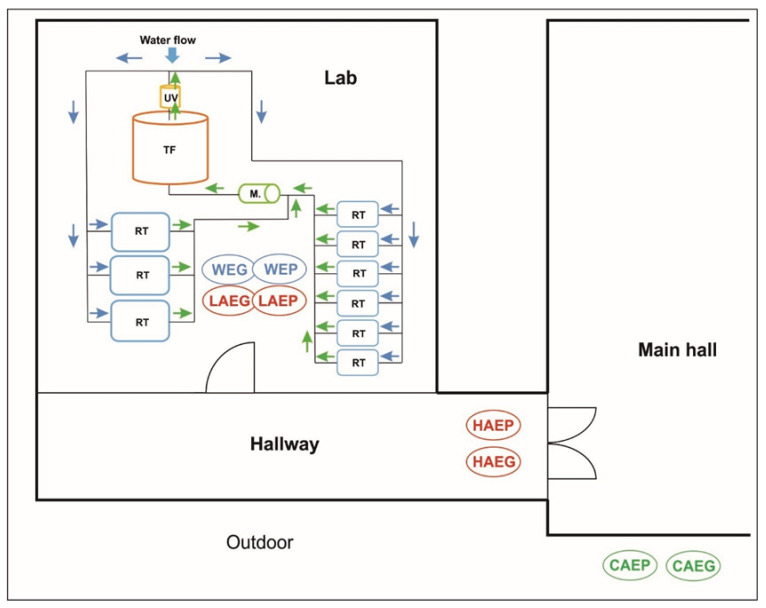
Indoor air and water sampling sites in the building of the Aquaculture and Ecological Engineering Center of the University of Warmia and Mazury in Olsztyn (Poland). Lab—laboratory; CAEG—control (outdoor) air sampled during the European grayling experiment; CAEP—control (outdoor) air sampled during the European perch experiment; LAEG—laboratory air sampled during the European grayling experiment; LAEP—laboratory air sampled during the European perch experiment; HAEG—hallway air sampled during the European grayling experiment; HAEP—hallway air sampled during the European perch experiment; WEG—water sampled from rearing tanks during the European grayling experiment; WEP—water sampled from rearing tanks during the European perch experiment; RT—rearing tank; M—microstrainer; TF—trickling filter; UV—UV lamp.

The locations of the air and water sampling sites are presented in Figure 1.

#### 2.2.1. Sampling Periods

Samples of indoor air from the laboratory (L) and the hallway (H), and water samples from the fish rearing tanks (FT) were collected three times (in monthly intervals) during the experimental rearing of each fish species. Air and water samples were collected between 15 April 2016 and 15 June 2016 (European grayling) and between 19 April 2019 and 19 June 2019 (European perch). During each rearing experiment, air and water samples were collected between 8 and 10 a.m., before fish feeding. 

#### 2.2.2. Air Sampling 

Aerosol samples were collected three times from all experimental (LAEG, LAEP, HAEG, and HAEP) and control (CAEG and CAEP) sites during the experimental rearing of each fish species (Figure 1). Air was sampled with the use of the MAS-100 Eco impaction air sampler (Merck) with 400 inlet orifices and a flow rate of 100 L/min. Air was aspirated onto a 90 mm contact Petri plate containing an agar medium for culturing *Aeromonas* spp. Airborne bacteria were transferred to the culture medium with an impaction speed of 11 m/s. The MAS-Eco device has a sampling volume of 1 to 1000 L. In the present study, the air sampling volume was 200 to 1000 L, depending on the site. During each rearing experiment, the air was sampled three times from each experimental site. 

Eighteen samples of indoor air and nine samples of outdoor air were collected during each rearing experiment. A total of 36 indoor air samples and 18 outdoor air samples were collected during the study. 

#### 2.2.3. Water Sampling

During each rearing experiment, water was sampled from 3 out of the 9 rearing tanks stocked with a given fish species. Nine water samples were collected from the European grayling tanks and 9 samples were collected from the European perch tanks. A total of 18 water samples for microbiological analyses were collected during the study. 

### 2.3. Microbiological Analyses

#### 2.3.1. *Aeromonas hydrophila* Counts in the Samples of Indoor Air and Tank Water 

The total counts of mesophilic *A. hydrophila* (TCMAH) in all air and water samples were determined on the Aeromonas Medium Base (Ryan) incubated at 37 °C for 24 h. The analyzed bacterial groups were quantified by the pour plate method. The TCMAH in the air and water samples were determined based on the number of opaque green colonies with a dark center that were formed on the Aeromonas Medium Base (Ryan). Next, mesophilic *A. hydrophila* strains were identified by fluorescence in situ hybridization (FISH) with a Cy3-labeled oligonucleotide probe (KO 229) [26]. The FISH protocol (including hybridization conditions) and the microscopic analysis of *A. hydrophila* isolates were described previously by Gołaś et al. [24]. Finally, the TCMAH were determined on the Aeromonas Medium Base (Ryan) based on the number of opaque green colonies with a dark center that were hybridized in the FISH assay with the KO 229 probe [26]. The *Aeromonas hydrophila* ATCC 7966 (American Type Culture Collection, Manassas, VA, USA) reference strain was used to control the oligonucleotide probe (KO 229) binding during in situ hybridization.

To determine the counts of potentially pathogenic *A. hydrophila* (PPAH), all mesophilic *A. hydrophila* strains (TCMAH) were cultured on TSA medium with 5% addition of sheep blood [27]. Based on the protocol developed by Hsu et al. [28], isolates exhibiting high hemolytic activity at R ≥ 4 were classified as PPAH. During the study, a total of 150 mesophilic *A. hydrophila* (TCMAH) strains and 93 PPAH strains were isolated from all air samples, and a total of 170 mesophilic *A. hydrophila* strains and 90 potentially pathogenic *A. hydrophila* (PPAH) strains were isolated from the water samples. 

In the samples of indoor (LAEG, LAEP, HAEG, and HAEP) and outdoor (CAEG and CAEP) air, the TCMAH and PPAH counts on the Aeromonas Medium Base (Ryan), expressed as colony-forming units (cfu), were corrected with the use of Feller’s statistical correction table. 

The corrected counts were converted to cfu per m^3^ of air (cfu/m^3^). In order to compare the abundance of the analyzed bacterial groups between the two environments (air and water), the bacterial counts obtained from cultured water samples were also converted to cfu/m^3^. 

#### 2.3.2. Antibiotic Resistance of PPAH in Samples of Indoor Air and Water

The antibiotic resistance of all 183 potentially pathogenic *A. hydrophila* (PPAH) isolates was determined on Mueller-Hinton agar with the use of the disc diffusion technique [29]. The analysis involved 7 antimicrobial drugs belonging to the most popular classes of antibiotics: cephalosporins, sulfonamides, aminoglycosides, tetracyclines, macrolides, fluoroquinolones, and chloramphenicol. Antibiotics were applied in standard therapeutic doses: cefotaxime (CTX)—30 µg, cotrimoxazole (SXT)—25 µg, gentamicin (CN)—10 µg, tetracycline (TE)—30 µg, erythromycin (E)—15 μg, norfloxacin (NOR)—10 µg, and chloramphenicol (C)—30 μg. Twenty-four-hour cultures of the bacterial strains were suspended in 0.85% saline solution, and the suspension turbidity was adjusted to 0.5 McFarland. The inocula were plated on Mueller–Hinton agar. After 30 min, discs saturated with antibiotics were applied to the plates with the use of a dispenser. Plates containing the antibiotic discs were incubated at 30°C. The antibiotic susceptibility of the tested strains was evaluated based on the guidelines of the Clinical and Laboratory Standards Institute [30]. The *Aeromonas hydrophila* ATCC 7966 (American Type Culture Collection, Manassas, VA, USA) reference strain was additionally used as the control microorganism to verify the antibacterial effect of the studied drugs [31]. Multidrug resistance was determined by calculating the MAR index based on the resistance of potentially pathogenic *A. hydrophila* (PPAH) isolates to a minimum of two antibiotics representing different classes, as described by Krumperman [32]. Natural environments where antibiotics are absent or are present only sporadically have an MAR value of ≤0.2, whereas environments at high risk of antibiotic exposure have an MAR value of >0.2 [33].

### 2.4. Physical Parameters of Air and Water Samples 

Two physical parameters were determined in the air and water samples collected during the fish rearing experiments. The temperature (°C) and relative humidity (%) were measured in the samples of indoor (LAEG, LAEP, HAEG, and HAEP) and outdoor (CAEG and CAEP) air. The temperature (°C) was measured in the water samples (WEG and WEP). In all of the air samples, the temperature and relative humidity were measured with an EBI 2-TH-611/6120 digital data logger with an accuracy of ±0.1 °C. The water temperature in the fish rearing tanks was measured with a WTW Multiline P4 multi-parameter sensor with an accuracy of ±0.1 °C.

### 2.5. Statistical Analysis

The presence of relationships between the PPAH counts and the physical parameters of the indoor air and water samples collected during the two fish rearing experiments was determined by principal component analysis (PCA) with the use of CANOCO 5.0 software [34]. All variables were standardized to zero mean and unit variance before PCA. Significant differences (*p* ≤ 0.05) in the proportion of PPAH in TCMAH between the air samples were determined in a two-tailed test with the use of XLSTAT, a statistical add-on for Microsoft Excel (Addinsoft). The similarity of the sampling sites was evaluated in cluster analysis (tree diagram, single linkage, and Euclidean distances). The distance between the clusters was measured with Ward’s method based on the proportion of potentially pathogenic *A. hydrophila* (PPAH), that were resistant to the standard therapeutic doses of the applied antibiotics (CTX, SXT, CN, TE, E, NOR, and C). Cluster analysis was conducted based on the criteria described by Sneath [35]. Data were processed statistically using the Statistica 13.3 program (StatSoft Inc. 1984–2017, TIBCO Software Inc., Palo Alto, CA, USA).

## 3. Results

### 3.1. Aeromonas hydrophila Counts in Air and Water Samples Collected during Fish Rearing Experiments

The indoor air sampled from various sites differed in the TCMAH and PPAH counts. The TCMAH and PPAH counts were lowest (from several to several dozen cfu/m^3^) in HAEG and HAEP, and the values noted in these samples were several—to ten-fold higher than those in the control samples of outdoor air (CAEG and CAEP). The TCMAH and PPAH counts were highest in the LAEG and LAEM samples collected in the area of the rearing tanks stocked with both fish species, and they ranged from around 2.3 × 10^2^ to 1.7 × 10^3^ cfu/m^3^ (TCMAH) and from 2.8 × 10^1^ to 6.9 × 10^2^ cfu/m^3^ (PPAH). In the samples of tank water (WEG and WEP), the counts of mesophilic *A. hydrophila* (TCMAH) and potentially pathogenic *A. hydrophila* (PPAH) were several orders of magnitude higher than those in the samples of indoor air. Based on the recommended values for hemolytic bacteria in homes and non-industrial indoor environments [36], the LAEG, LAEP, HAEG, and HAEP samples were characterized by low (<100 cfu/m^3^) or moderate (100–500 cfu/m^3^) bacterial contamination. The I/O index was high in the range of 16.98–481.38 (LAEG and LAEP) and 3.35–65.26 (HAEG and HAEP) (Table 1).

**Figure 2:**
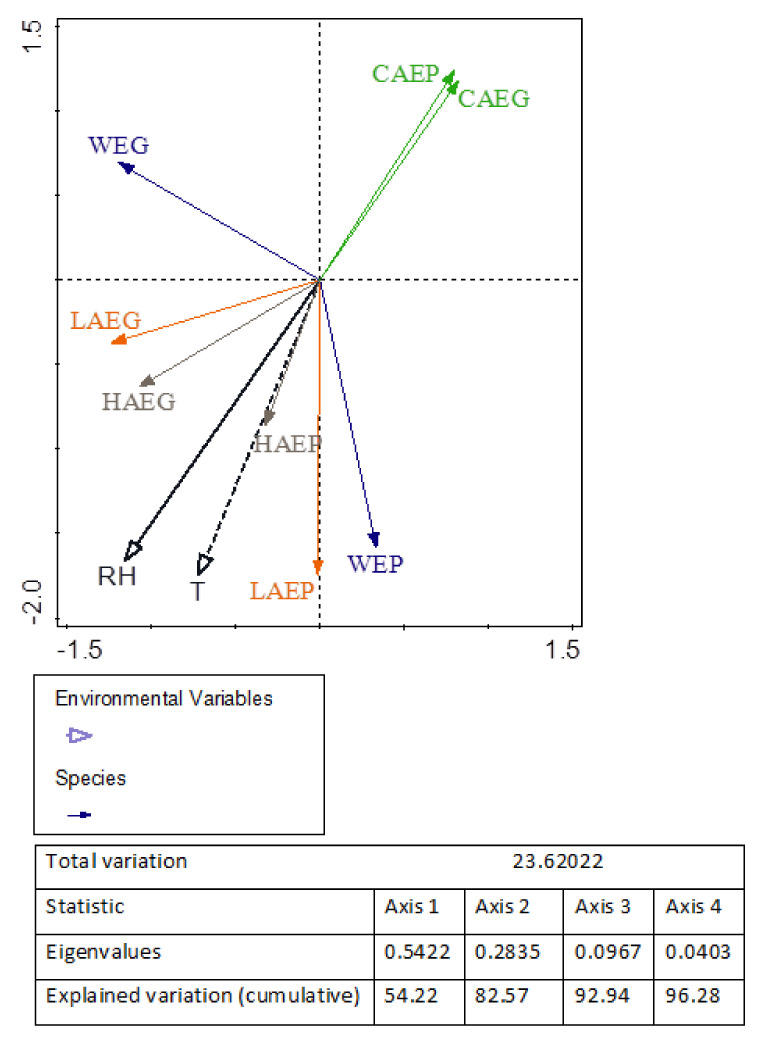
PCA biplot of the correlations (*p* ≤ 0.05) between the counts of potentially pathogenic *Aeromonas hydrophila* (PPAH) in the samples of indoor air and water collected during the European grayling (LAEG, HAEG, CAEG, and WEG) and European perch (LAEP, HAEP, CAEP, and WEP) experiments. Yellow lines denote the bacterial counts in the samples of laboratory air (LAEG and LAEP); grey lines denote the bacterial counts in the samples of hallway air (HAEG and HAEP); green lines denote the bacterial counts in the control samples of outdoor air (CAEG and CAEP); blue lines denote the bacterial counts in the water samples (WEG and WEP); and black lines denote the physical parameters: R—relative humidity (solid line) and T—temperature (dashed line).

The unconstrained PCA revealed strong positive relationships between the PPAH counts in the air samples collected from the laboratory (LAEG and LAEP) where two fish species were reared and the water samples (WEG and WEP). Positive correlations were also observed between the PPAH counts in the air samples collected in the laboratory (LAEG) and the hallway (HAEG) during the European grayling experiment (Figure 2).

**Figure 3:**
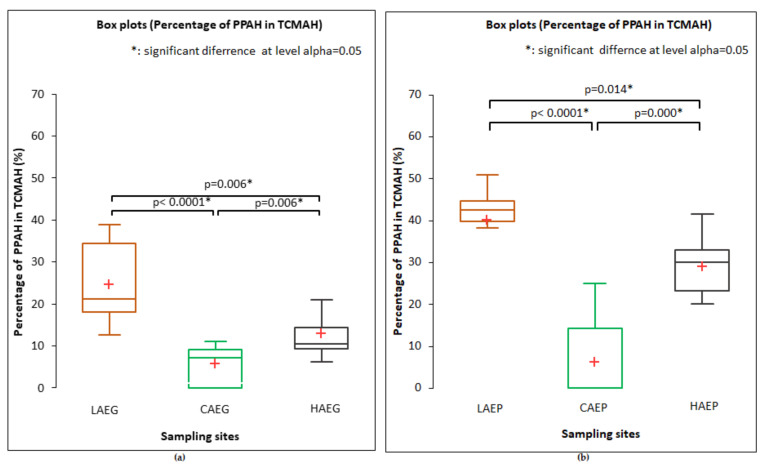
Differences in the proportion of potentially pathogenic *Aeromonas hydrophila* (PPAH) in the total counts of mesophilic *Aeromonas hydrophila* (TCMAH) in the samples of indoor (LAEG, HAEG, LAEP, and HAEP) and outdoor (CAEG and CAEP) air collected during the rearing experiments involving (**a**) European grayling, and (**b**) European perch, determined by the two-tailed test. CAEG—control (outdoor) air during the European grayling experiment; CAEP—control (outdoor) air during the European perch experiment; LAEG—laboratory air during the European grayling experiment; LAEP—laboratory air during the European perch experiment; HAEG—hallway air during the European grayling experiment; and HAEP—hallway air during the European perch experiment. Error bars denote the minimum/maximum values. The red plus sign denotes the mean value.

In the samples of indoor air, the proportion of PPAH in TCMAH was highest in LAEG (24.5 ± 8.9%) and LAEP (40.1 ± 9.5%) during both rearing experiments. The above parameter ranged from 7.4% to 41.7% in the air samples collected in the hallway (HAEG and HAEP). In the CAEG and CAEP samples, the proportion of PPAH in TCMAH was low, in the range of 0–9.5%. The two-tailed test (t-test for two independent samples) revealed significant (*p* ≤ 0.05) differences in the proportion of PPAH in TCMAH between the samples of indoor air collected during the European grayling experiment and the European perch experiment (Figure 3a,b).

### 3.2. Physical Parameters and Their Influence on the Counts of Potentially Pathogenic A. hydrophila in Samples of Indoor Air 

The temperature and relative humidity of the air samples and the temperature of the water in the fish rearing tanks are presented in Table 2. In the samples of outdoor air (CAEG and CAEP), the temperature was determined to be in a range of 17.2–25.8 °C, and the relative humidity was in a range of 27.8–32.4%. Similar temperatures (20.0–22.4 °C) and relative humidities (30.6–34.8%) were determined in the HAEG and HAEP samples. The temperature of the indoor air samples ranged from 12.2 °C (LAEG) to 18.3 °C (LAEP), and the relative humidity ranged from 41.4% (LAEG) to 49.2% (LAEP). The water temperature in rearing tanks was optimal for the evaluated fish species, and it was determined to be 10.8–11.2 °C (WEG) and 17.5–18.5 °C (WEP). Unconstrained PCA revealed significant positive relationships (*p* ≤ 0.05; N = 27) between the PPAH counts vs. temperature and relative humidity in all indoor samples (Figure 2). 

### 3.3. Antibiotic Resistance of Potentially Pathogenic A. hydrophila

**Figure 4:**
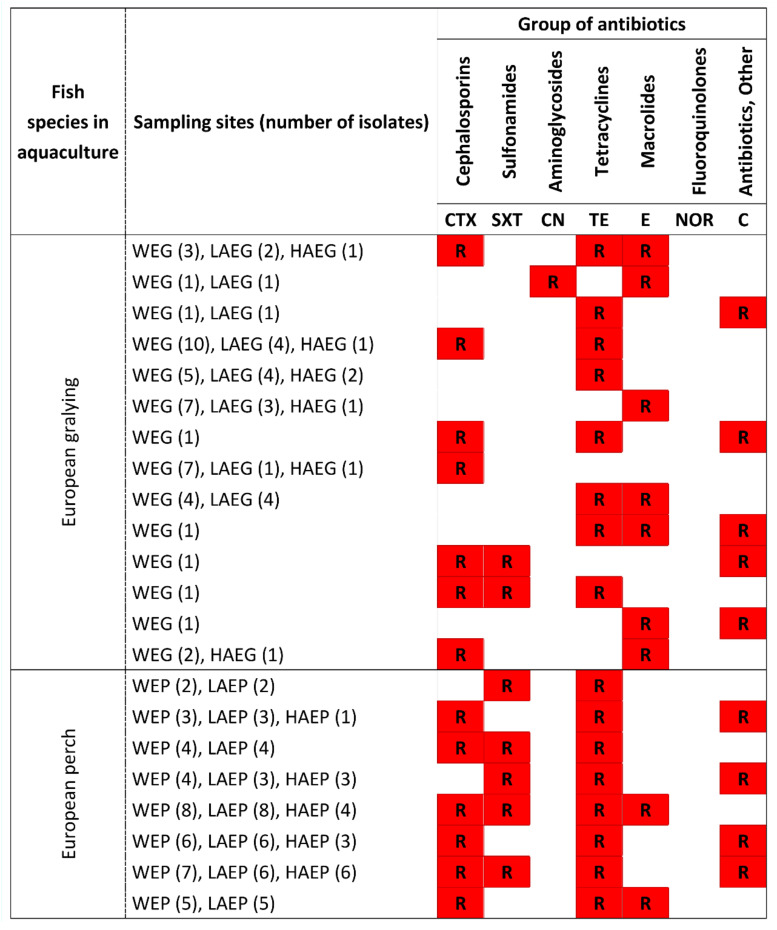
Antibiotic resistance profile of potentially pathogenic *Aeromonas hydrophila* (PPAH) strains isolated from the indoor air (LAEG, HAEG, LAEP, and HAEP), outdoor air (CAEG and CAEP), and water (WEG and WEP) during the fish rearing experiments in RAS. CAEG—control (outdoor) air during the European grayling experiment; CAEP—control (outdoor) air during the European perch experiment; LAEG—laboratory air during the European grayling experiment; LAEP—laboratory air during the European perch experiment; HAEG—hallway air during the European grayling experiment; HAEP—hallway air during the European perch experiment; WEG—water collected from rearing tanks stocked with European grayling; WEP—water collected from rearing tanks stocked with European perch. The number of isolated strains is given in brackets. Red squares with the letter “R” denote antibiotic resistance.

**Figure 5:**
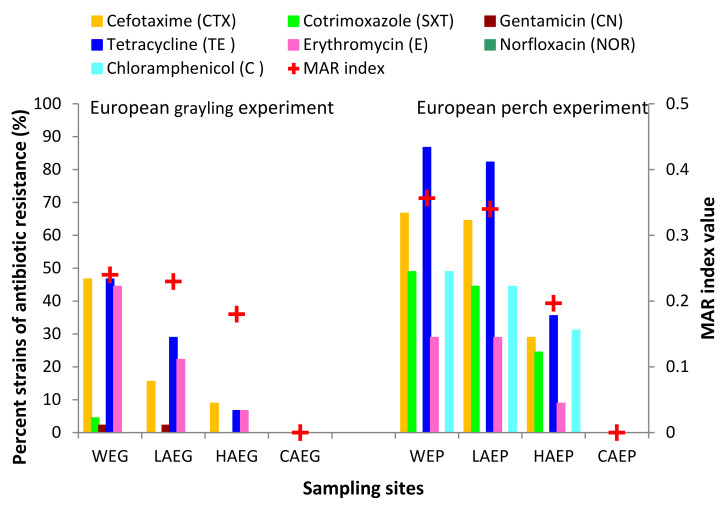
Antibiotic resistance of potentially pathogenic Aeromonas hydrophila (PPAH) strains isolated from the samples of indoor air (LAEG, HAEG, LAEP, and HAEP), outdoor air (CAEG and CAEP), and water (WEG and WEP) during the rearing experiments involving European grayling and European perch. The red plus sign denotes the MAR index.

The results of the analysis evaluating the resistance of airborne and waterborne PPAH strains to standard therapeutic doses of cefotaxime (CTX), cotrimoxazole (SXT), gentamicin (CN), tetracycline (TE), erythromycin (E), norfloxacin (NOR), and chloramphenicol (C) are presented in Figure 4. All strains isolated from the control samples of outdoor air (CAEG and CAEP) were susceptible to all of the tested antibiotics. In the samples of indoor air, the highest percentage of strains that were resistant to the analyzed antibiotics was noted in laboratory air (LAEG and LAEP) during the experiments involving both fish species. In the total number of 90 PPAH strains isolated from LAEG and LAEP, 44.4% and 82.2% of the strains, respectively, were resistant to the tested antibiotics. In the PPAH isolates from WEG and WEP, 86.7% to 95.5% of the strains were resistant to the analyzed antimicrobials. In the samples of hallway air, 15.5% and 37.7% of the strains isolated from HAEG and HAEP, respectively, were resistant to the standard therapeutic doses of the analyzed antibiotics. Most strains isolated from the indoor air and water were resistant to CTX and TE, and fewer strains were resistant to SXT, E, and C. In the WEG and WEP samples, 55.5–73.3% (25–33 isolates) and 57.7–86.7% (26–39 isolates) of the isolated strains were resistant to CTX and TE, respectively. In the LAEG and LAEP samples, resistance to TE and CTX was determined in 33.3–82.2% (15–37 isolates) and 15.6–73.3% (7–33 isolates) of the isolated strains, respectively. Resistance to SXT, E, and C was noted in 0.0–44.4% (0–21 isolates) of the strains isolated from WEG, WEP, LAEG, and LAEP. Potentially pathogenic *A. hydrophila* strains isolated from HAEG and HAEP were also resistant to TE, C, CTX, SXT, and E. Resistance to these antibiotics was determined in 6.7–35.6%, 0.0–31.1%, 8.9–28.9%, 0.0–24.4%, and 0.0–8.9% of the isolates. Regardless of the reared fish species, the strains isolated from laboratory indoor air (LAEG and LAEP) had identical antibiotic resistance profiles to the strains isolated from water (WEG and WEP). Most strains isolated from the water (WEG and WEP) and indoor air (LAEG and LAEP) were also resistant to multiple drugs (at least two antibiotics from different classes). The nominal values of the MAR index were highest in the PPAH strains isolated from the rearing tanks (WEG and WEP) and laboratory air (LAEG and LAEP). The MAR index was determined to be 0.24–0.36 in the water samples (WEG and WEP), 0.23–0.34 in the laboratory air samples (LAEG and LAEP), and 0.0 in the outdoor air samples (CAEG and CAEP) (Figure 5). 

**Figure 6:**
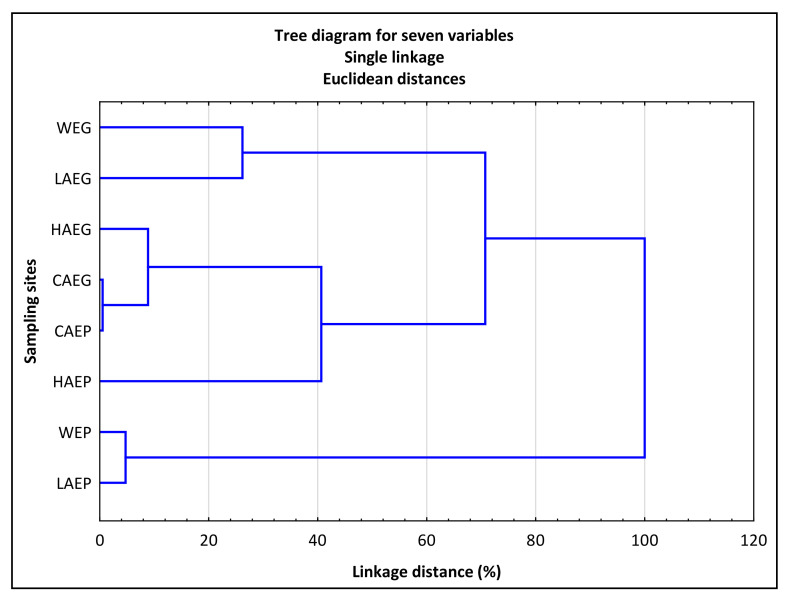
Similarities between the indoor air and water sampling sites based on the counts of potentially pathogenic *Aeromonas hydrophila* (PPAH) resistant to cefotaxime (CTX), cotrimoxazole (SXT), gentamicin (CN), tetracycline (TE), erythromycin (E), norfloxacin (NOR), and chloramphenicol (C). CAEP and CAEP—control samples of outdoor air collected during the European grayling and European perch experiments, respectively; LAEG and LAEP—samples of laboratory air collected during the European grayling and European perch experiments, respectively; HAEG and HAEP—samples of hallway air collected during the European grayling and European perch experiments, respectively; WEG and WEP—samples of tank water collected during the European grayling and European perch experiments, respectively.

The analyzed air and water samples from different sites were evaluated for similarity by agglomerative clustering based on the counts of potentially pathogenic *A. hydrophila* (PPAH) resistant to CTX, SXT, CN, TE, E, NOR, and C (Figure 6). The greatest similarities were noted in the first cluster composed of strains isolated from the indoor air (LAEP) and water (WEP) during the European perch experiment. The second cluster contained PPAH strains isolated from the indoor air (LAEG) and water (WEG) during the European grayling experiment. The third cluster comprised strains isolated from the indoor and outdoor air that formed a network of interconnected nodes. In the third cluster, the greatest similarities were observed between the PPAH strains isolated from the control samples of outdoor air (CAEG and CAEP). The greatest differences were noted in the antibiotic-resistant PPAH isolated from HAEG during the European grayling experiment (Figure 6).

## 4. Discussion

High microbial contamination of indoor air increases the risk of certain health problems for the occupants. The type and range of indoor activities are chiefly responsible for the deterioration in air quality in workplaces [2,6]. Human activities influence the microbial levels in bioaerosols, and they can increase the concentrations of potentially pathogenic microorganisms, including those that originate from the aquatic environment [19,37]. Bioaerosols, which are formed due to the emission of small water droplets, can harbor Gram-negative waterborne bacteria [18]. Bioaerosols can be also a source of *A. hydrophila*, an autochthonous bacterial species that is ubiquitous in aquatic environments [15,38]. This bacterial species occurs naturally in fish microbiota and water, and it widely colonizes industrial fish farms and experimental aquaculture [16,17,39].

In the current study, considerable differences in the counts of potentially pathogenic *A. hydrophila* (PPAH) were noted in the samples of indoor air collected during the experimental rearing of European grayling and European perch. However, even the highest PPAH counts in indoor aerosols (LAEG, HAEG, LAEP, HAEP) did not exceed 5.0 × 10^3^ cfu/m^3^, which is the maximum permissible limit of contamination with mesophilic bacteria in public buildings [6]. Based on the guideline values for hemolytic bacteria in homes and non-industrial indoor environments [36], the analyzed samples of LAEG, LAEP, HAEG, and HAEP were characterized by low (<100 cfu/m^3^) to moderate (100–500 cfu/m^3^) levels of bacterial contamination. Regardless of the reared fish species, the PPAH counts were highest in the samples of indoor air collected from the laboratory (LAEG and LAEP) where the fish were reared. The concentrations of these potential pathogens were 10 to 100 lower in the control samples of outdoor air (CAEG and CAEP). 

According to the hygiene standards developed by the American Industrial Hygiene Association [40], acceptable limits of microbial contamination are determined by calculating the ratio of bacterial counts in indoor aerosols (I) to outdoor air (O), and the results are used to identify the main source of contamination. An indoor/outdoor (I/O) ratio below 1 (I/O < 1) is generally indicative of the absence of indoor contamination or acceptable contamination levels. In turn, high values of the I/O ratio point to an indoor source of microbial emissions. The above guidelines were applied to interpret the total counts of mesophilic *A. hydrophila* (TCMAH) and potentially pathogenic *A. hydrophila* (PPAH) counts in the samples of indoor air, and the analysis revealed very high values of the I/O ratio in indoor facilities in the ranges of 3.35–481.38 and 4.56–400.00, respectively (Table 1). These results indicate that PPAH were the main source of air contamination in the laboratory and the hallway during both rearing experiments. The TCMAH and PPAH counts were high in the samples of tank water (1.3 × 10^9^–8.5 × 10^9^ and 0.4 × 10^9^–3.9 × 10^9^ cfu/m^3^, respectively), and, due to the fish activity in tanks and the resulting movement of water, these bacteria were transmitted by water droplets to laboratory air. Therefore, the water in the tanks where both fish species were reared was the main source of the analyzed bacteria. The influence of the aquatic environment on the contamination of laboratory air (LAEG and LAEP) with potentially pathogenic *A. hydrophila* (PPAH) was confirmed by PCA, which revealed strong positive relationships (*p* ≤ 0.05) between the PPAH counts in samples of water (WEG and WEP) and indoor air collected from the laboratory (LAEG and LAEP) where both fish species were experimentally reared. An increase in the microbial contamination of indoor air was also positively correlated (PCA, *p* ≤ 0.05) with temperature and relative humidity. Similar observations were made by Li et al. [13] and Kalogerakis et al. [41], who analyzed samples of indoor air from other public utility buildings and concluded that the concentrations of airborne microorganisms increased with a rise in the relative humidity.

The antibiotic resistance profile of the analyzed potentially pathogenic *A. hydrophila* (PPAH) confirmed that the rearing environment of European grayling and European perch had the potential to cause adverse health effects in humans. Bacterial strains isolated from samples of indoor air (LAEG, LAEP, HAEG, and HAEP), outdoor air (CAEG and CAEP), and tank water (WEG and WEP) were analyzed for sensitivity to standard therapeutic doses of seven antibiotics belonging to different classes (CTX, SXT, CN, TE, E, NOR, and C). The analysis revealed that all strains isolated from the CAEG and CAEP samples were susceptible to all of the tested antibiotics. In the group of the studied PPAH monocultures, 44.4–82.2% of the strains isolated from LAEG and LEAP and 86.6–97.8% of the strains isolated from WEG and WEP were resistant to antimicrobials. In the rearing experiments conducted on both fish species, most of the isolates from the samples of indoor air and tank water were resistant to TE and CTX, and some strains were also resistant to standard therapeutic doses of SXT, E, and C. Resistance to TE was noted in 57.7–86.7%, 33.3–82.2%, and 8.9–37.7% of the strains isolated from water (WEG and WEP), laboratory air (LAEG and LAEP), and hallway air (HAEG and HAEP), respectively. Resistance to CTX was observed in 55.5–73.3%, 15.6–73.3%, and 8.9–31.1% of PPAH sampled from tank water (WEG and WEP), laboratory air (LAEG and LAEP), and hallway air (HAEG and HAEP), respectively. Resistance to SXT, E, and C was noted in 0.0–44.4% of the isolates from tank water and indoor air. The antibiotic resistance profiles of *A. hydrophila* isolated from indoor and outdoor air have never been described in the literature, because this bacterial species is not considered in studies monitoring the microbiological quality of indoor and outdoor air. It should also be noted that indoor air microbiota comprise mainly Gram-positive bacteria, whereas Gram-negative bacteria, including *A. hydrophila*, are sporadically detected [7,42,43]. Only a single species of Gram-positive bacteria was identified in a study analyzing the antibiotic resistance of strains isolated from bioaerosols in an office building in Gliwice, Poland. The identified species was *Pseudomonas putida*, which is resistant to penicillins, sulfonamides, tetracyclines, and other antibiotics [7]. However, research studies evaluating the efficacy of various antimicrobials against *A. hydrophila* strains isolated from aquaculture demonstrated differences in the antibiotic resistance of the isolates depending on the fish species, stage of development, fish rearing system, and climate. In a study of *A. hydrophila* isolated from fish ponds in semi-intensive aquaculture in the late stage of the carp fattening cycle, 95%, 65%, and 15% of the isolates were resistant to trimethoprim, tetracycline, and chloramphenicol, respectively. Monir et al. [44] isolated *A. hydrophila* strains from air-breathing magur catfish (*Clarias batrachus*) in aquaculture and found that 100% of the isolates were resistant to ampicillin, 96% were resistant to penicillin, and 92% were resistant to novobiocin. *Aeromonas hydrophila* strains isolated from intensive aquaculture in the Southeast Region of Brazil were moderately resistant to amoxicillin, ampicillin, lincomycin, novobiocin, oxacillin, penicillin, and tetracycline [45]. The observed variations in the antibiotic resistance of environmental bacteria can be attributed not only to direct exposure to antimicrobials, but also to the acquisition of resistance from clinical strains [46]. In aquatic environments colonized by human and animal bacterial pathogens, resistance to antimicrobials can be spread by horizontal gene transfer between waterborne bacteria and other environmental bacteria [47]. In a study by Pepi and Focardi [48], 90% of waterborne bacteria were resistant to at least one antibiotic, and around 20% of the strains were resistant to multiple antimicrobials.

In the present study, most strains isolated from the indoor air and tank water had similar antibiotic resistance profiles, regardless of the reared fish species. The majority of strains isolated from the indoor air and tank water were resistant to two or more antibiotics. During the European grayling experiment, most of the isolated PPAH strains were resistant to CTX + TE, TE, and E, whereas most of the strains isolated during the European perch experiment were resistant to three or four antibiotics. The increase in the antibiotic resistance spectrum of potentially pathogenic *A. hydrophila* (PPAH) strains could have resulted from the higher temperature of water (18 °C ± 0.5) and indoor air (17.9 °C ± 0.4 to 20.2 °C ± 0.2) during the European perch rearing than during the European grayling experiment. This observation is consistent with the findings of Pepi and Focardi [48], which indicate that antibiotic resistance is influenced by temperature and that the resulting changes are maintained in the microbial community. At higher temperatures, some bacterial species can produce a biofilm at the air-liquid interface or undergo mutations that enable them to develop antibiotic resistance. These adaptations can increase their tolerance to many antibiotics and lead to multidrug resistance.

In the group of 180 PPAH strains isolated from indoor air, 54.4% of multidrug-resistant strains (resistant to at least two antibiotics belonging to different classes) were isolated from the laboratory (LAEG and LAEP) where both fish species were experimentally reared. These findings were validated by the calculated values of the MAR index, which were higher (0.22–0.34) in strains isolated from the laboratory than in strains isolated from hallway air (HAEG and HAEP; MAR = 0.04–0.20) and outdoor air (CAEG and CAEP; MAR = 0.00). Most potentially pathogenic *A. hydrophila* (PPAH) strains originated from the indoor air samples collected in the laboratory (LAEG and LAEP), a site where the risk of microbial contamination is high and where antibiotics are frequently used, which confirms the hypothesis formulated by Paul et al. [33]. Moreover, the MAR index of PPAH strains isolated from LAEG and LAEG approximated nominal MAR values (0.24–0.36) determined in the strains from WEG and WEP, which indicates that the tank water was the main source of contamination with multidrug-resistant waterborne strains that were transmitted to indoor air. The cluster analysis confirmed close similarities between the potentially pathogenic *A. hydrophila* isolates. Strains resistant to the tested antibiotics were divided into three clusters. The first cluster comprised PPAH strains isolated from WEG and LAEG, the second cluster was composed of isolates from WEP and LAEP, and the third cluster contained PPAH strains isolated from hallway air (HAEP and HAEG) and outdoor air (CAEG and CAEP).

The observed increase in the counts of multidrug-resistant PPAH strains in indoor air suggests that aquaculture environments can act as hot spots for the airborne transmission of antibiotic-resistant strains due to antibiotic selection pressure resulting from the excessive use of antimicrobials and/or direct exposure to other drug-resistant strains [49]. Bacteria of the genus *Aeromonas* are known for their enhanced capacity to acquire and exchange antibiotic resistance genes with other microorganisms, including enterobacteria. Therefore, the transmission of antibiotic-resistant pathogens from aquatic environments to air can pose new health risks for humans, even at the microscale, as demonstrated by the present experiment.

## 5. Conclusions

In this study, the counts of potentially pathogenic *A. hydrophila* were low (<100 cfu/m^3^) to moderate (100–500 cfu/m^3^) in the analyzed samples of indoor air. In the air samples collected from the laboratory (LAEG, LAEP), where rearing experiments were conducted on European grayling and European perch, the counts of potentially pathogenic *A. hydrophila* (PPAH) were several-fold higher than those in the outdoor air samples (CAEG and CAEP). The very high values of the I/O ratio of bacterial counts in indoor aerosols (I) to outdoor air (O) indicated that the fish rearing tanks were the main internal source of PPAH. The bacterial strains isolated from laboratory air (LAEG and LAEP) were examined for resistance to seven popular antibiotics, and the analysis revealed that the laboratory employees were at a higher risk of infection. Most potentially pathogenic *A. hydrophila* strains isolated from the laboratory air (LAEG and LAEP) were characterized by multidrug resistance (MAR > 0.20). The antibiotic resistance profiles of most PPAH isolates from the laboratory bioaerosols (LAEG and LAEP) were similar to the profiles of PPAH strains isolated from the water in the rearing tanks stocked with European grayling (WEG) and European perch (WEP). The similarities between the potentially pathogenic *A. hydrophila* strains isolated from the water and indoor air were confirmed by cluster analysis, where the analyzed strains formed three distinctive clusters. The first cluster comprised PPAH strains isolated from WEG and LAEG, the second cluster contained isolates from WEP and LAEP, and the third cluster was composed of strains isolated from the outdoor air. The results of this study indicate that aquaculture can act as hot spots for airborne transmission of antibiotic-resistant and potentially pathogenic *A. hydrophila*.

## Figures and Tables

**Table 1:** Total counts of mesophilic *A. hydrophila* (TCMAH) and counts of potentially pathogenic *A. hydrophila* (PPAH) in the air and water samples collected during the European grayling and European perch rearing experiments conducted in a recirculating aquaculture system.

Sample	Bacterial Counts (cfu/m^3^)		Indoor/Outdoor Ratio (I/O)	
	TCMAH ^1^	PPAH ^2^	TCMAH	PPAH
CAEG ^3^	0.8 × 10^1^ ± 0.3 × 10^1^	0.2 × 10^1^ ± 0.1 × 10^1^	na ^11^	na
LAEG ^4^	5.3 × 10^2^ ± 3.0 × 10^2^	4.3 × 10^1^ ± 1.5 × 10^1^	16.98	38.33
HAEG ^5^	3.2 × 10^1^ ± 0.1 × 10^1^	0.5 × 10^1^ ± 0.3 × 10^1^	3.35	4.56
CAEP ^6^	0.3 × 10^1^ ± 0.3 × 10^1^	0.1 × 10^1^ ± 0.1 × 10^1^	na	na
LAEP ^7^	1.4 × 10^3^ ± 0.3 × 10^3^	4.7 × 10^2^ ± 2.2 × 10^2^	481.38	400.00
HAEP ^8^	9.2 × 10^1^ ± 6.5 × 10^1^	4.0 × 10^1^ ± 1.0 × 10^1^	65.26	35.00
WEG ^9^	3.3 × 10^9^ ± 2.0 × 10^9^	0.9 × 10^9^ ± 0.5 × 10^9^	na	na
WEP ^10^	7.0 × 10^9^ ± 1.5 × 10^9^	2.8 × 10^9^ ± 1.1 × 10^9^	na	na

^1^—total counts of mesophilic *A. hydrophila*; ^2^—potentially pathogenic *A. hydrophila*; ^3^—control (outdoor) air during the European grayling experiment; ^4^—laboratory air during the European grayling experiment; ^5^—hallway air during the European grayling experiment; ^6^—control (outdoor) air during the European perch experiment; ^7^—laboratory air during the European perch experiment; ^8^—hallway air during the European perch experiment; ^9^—water collected from the rearing tanks during the European grayling experiment; ^10^—water collected from the rearing tanks during the European perch experiment; ^11^—not applicable.

**Table 2:** Temperature and relative humidity (mean values and standard deviation) of the air and water samples collected during European grayling and European perch rearing experiments.

Sample	Temperature (°C)	Relative Humidity (%)
CAEG ^1^	20.9 ± 3.5	30.1 ± 2.3
LAEG ^2^	12.5 ± 0.3	41.9 ± 0.5
HAEG ^3^	21.9 ± 0.5	32.7 ± 2.1
CAEP ^4^	21.5 ± 4.3	31.0 ± 0.8
LAEP ^5^	17.9 ± 0.4	48.3 ± 0.9
HAEP ^6^	20.2 ± 0.2	33.0 ± 0.5
WEG ^7^	11.0 ± 0.2	na ^9^
WEP ^8^	18.0 ± 0.5	na

^1^—control (outdoor) air sampled during the European grayling experiment; ^2^—laboratory air sampled during the European grayling experiment; ^3^—hallway air sampled during the European grayling experiment; ^4^—control (outdoor) sampled air during the European perch experiment; ^5^—laboratory air sampled during the European perch experiment; ^6^—hallway air sampled during the European perch experiment; ^7^—water sampled from rearing tanks during the European grayling experiment; ^8^—water sampled from rearing tanks during the European perch experiment; ^9^—not applicable.

## Data Availability

The data presented in this study are available on request from the corresponding author.
